# Are cash incentives always king? A randomized controlled trial evaluating hedonic versus cash incentives (TEH-C)

**DOI:** 10.3389/fpubh.2024.1354814

**Published:** 2024-04-30

**Authors:** Eric Andrew Finkelstein, Michelle Tian Nee Chow, Mihir Gandhi

**Affiliations:** ^1^Health Services & Systems Research Program, Duke-NUS Medical School, Singapore, Singapore; ^2^Centre for Quantitative Medicine, Duke-NUS Medical School, Singapore, Singapore; ^3^Department of Biostatistics, Singapore Clinical Research Institute, Singapore, Singapore; ^4^Tampere Center for Child, Adolescent, and Maternal Health Research: Global Health Group, Tampere University, Tampere, Finland

**Keywords:** incentives, rewards, cash, hedonic, randomized controlled trial, steps, accelerometer, physical activity

## Abstract

**Introduction:**

Physical inactivity is a risk factor for obesity and non-communicable diseases. Despite myriad health and non-health benefits resulting from physical activity (PA), most individuals do not meet PA recommendations. Providing an incentive for meeting activity goals may increase activity levels. Classical economists argue that cash is the best incentive. Behavioral economists have posited that hedonic (pleasurable) incentives (e.g., massages, restaurant meals) may be superior to cash when incentives are offered over multiple time periods. To date, no studies have directly compared the effectiveness of cash versus hedonic incentives in promoting PA across multiple time periods.

**Methods:**

We conducted a two-arm, parallel, 4-month randomized controlled trial with healthy adults in Singapore where participants were randomized to either cash or hedonic incentives. Participants could earn up to SGD50 (≈USD37) in cash or hedonic incentives each month they met the study’s step target of 10,000 steps daily on at least 20/25 days out of the first 28 days of a month. The primary objective was to compare the mean proportion of months that participants met the step target between the two arms.

**Results:**

By month 4, participants in the cash (*N* = 154) and hedonic incentive (*N* = 156) arms increased their mean daily steps by 870 (*p* < 0.001) and 1,000 steps (*p* < 0.001), respectively. The mean proportion of months the step target was achieved was 90.53 and 88.34 for participants in the cash and hedonic incentive arms respectively, but differences across arms were small and not statistically significant for this or any outcome assessed.

**Conclusion:**

Our findings suggest that both cash and hedonic incentives are effective at promoting physical activity but that neither strategy is clearly superior.

**Clinical trial registration**: ClinicalTrials.gov, NCT 04618757 registered on November 6, 2020.

## Introduction

Physical inactivity is a risk factor for obesity and non-communicable diseases (NCDs). It is also a primary driver for rising health expenditures and increased productivity losses (1, 2). By 2030, it is estimated that inactivity will be responsible for INT$520 billion in healthcare expenditures worldwide (3) and INT$34.5 billion due to lost productivity among employees (2).

Despite myriad health and non-health benefits resulting from physical activity (PA) (4), most individuals do not meet PA recommendations (4, 5). One way to increase activity levels is to provide an incentive for meeting activity goals. Classical economic theory suggests that low levels of activity result because individuals do not see the benefits (many of which accrue well into the future) as large enough to offset the immediate costs of the activity, including opportunity costs that may come with forgone earnings or lost leisure time. Incentivizing PA raises the immediate benefits. As shown in several studies, if the incentives are large enough, they will induce greater levels of activity (6–10). If this translates into sustained health improvements, incentives could be cost-effective or even cost saving.

Classical economists argue that cash is the best incentive because it is completely fungible: it could be converted to any non-cash equivalent (11, 12). However, some behavioral economists have posited that hedonic (pleasurable) incentives may be superior to cash when incentives are offered over multiple time periods [11, 13, 14 (Tournament 2)]. The theory of mental accounting explains this hypothesis (15, 16). According to this theory, individuals tend to classify money in a ‘cash earnings’ account and are prone to spend the earnings on utilitarian items (e.g., groceries, bills) that may provide only low increases in marginal utility. In contrast, hedonic incentives (e.g., massages, movie theater tickets, restaurant meals), when earned, force individuals to apply the incentives to something pleasurable, which has the potential to generate a greater increase in utility than their cash equivalent (11, 17). Hedonic incentives may also trigger positive affect and memories (11, 18). As a result, greater effort may be exerted to earn the incentives in subsequent periods as compared to their cash equivalent (11).

To date, no studies have directly compared the effectiveness of cash versus hedonic incentives in promoting PA across multiple time periods in efforts to test this hypothesis. That is the focus of this effort. We conducted a two-arm individual level randomized controlled trial to test whether modest hedonic incentives are more effective than their cash equivalent in motivating participants to meet monthly step targets over a 4-month period. Our primary hypothesis is that the mean proportion of months that participants meet the step target will be greater in the hedonic incentive arm compared the equivalent cash incentive arm. We also expect mean daily steps and Fitbit® fairly and very active minutes each month to be greater in the hedonic incentive arm compared to the cash incentive arm.

## Methods

### Study design, recruitment, and participant characteristics

**Figure 1:**
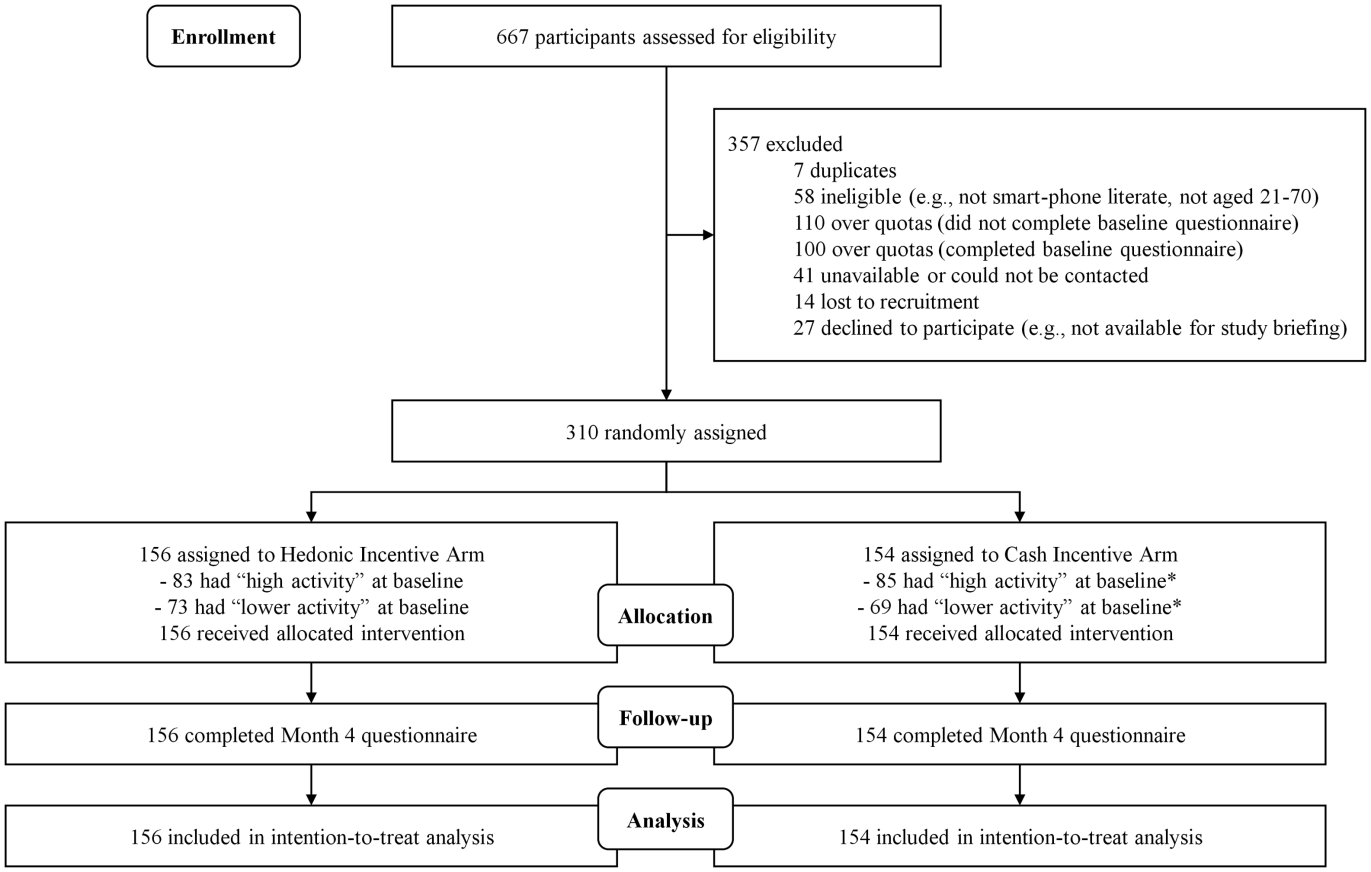
CONSORT diagram of TEH-C study participants. *Protocol deviation occured during randomization as 2 participants were randomized incorrectly as ‘lower activity’ due to oversight/technical errors. Figures shown correctly include the 2 participants in the 'high activity' at baseline group.

TEH-C (*T*rial *E*valuating *H*edonic versus *C*ash incentives) was a two-arm, 4-month single-blind randomized controlled trial comparing two parallel arms (1:1 allocation ratio): (1) cash incentive, and (2) hedonic incentives. The trial is registered on ClinicalTrials.gov (NCT 04618757). This manuscript conforms to CONSORT reporting guidelines (Figure 1).

General population participants in Singapore were directly recruited via flyers and online advertisements posted on social media platforms (i.e., Facebook and Instagram). All interested participants were directed to the study’s website to view more information about the study and to assess eligibility via a screening questionnaire (Screener Questionnaire, Supplementary material). We focused on Singapore’s population given that there is a growing emphasis on interventions that can cost-effectively decrease the onset of NCDs. Despite being a highly walkable city with numerous subsidized community-based physical activity programs, 25.1% of Singaporeans are insufficiently active (18). Incentivized physical activity programs have been shown to be effective on an individual level (6, 7) and population level (19). Thus, this population is well-suited for comparing the effectiveness of differing incentive strategies.

Participants were eligible if they were aged between 21 to 70 years, non-pregnant, residing in Singapore, English-speaking, and smartphone-literate. Eligible participants also had to be able to walk up 10 steps without resting, be willing to be randomly assigned to one of the intervention arms and wear a Fitbit® during waking hours throughout the study. Initially, we had used the Physical Activity Readiness Questionnaire (PAR-Q) (20) as screening tool to assess an individual’s readiness to safely engage in physical activity. Participants who answered ‘Yes’ to any of the questions on the PAR-Q (20), which suggests possible risk from increased activity, were deemed ‘conditionally eligible’ and required written doctor’s approval to confirm eligibility. However, this was subsequently changed such that participants who report being on doctor’s advice against engaging in moderate-to-vigorous physical activity (MVPA) and/or having a condition that restricts them from engaging in MVPA were deemed ‘ineligible’. All others were eligible to enroll.

All interested and eligible participants signed an informed consent document and an oath declaring that they will not cheat when striving to achieve the monthly step goal and that all the incentives they may earn will be obtained solely through their own efforts. This was done to minimize cheating (21). Participants then paid a non-refundable enrolment fee of SGD20 (≈USD14), which was set in place to deter those who were solely motivated to obtain the free activity tracker and were not truly interested in changing their behavior. The enrolment fee amount was determined to be a reasonable amount based on similar activity tracker-based studies in Singapore (6, 7) and was approved by the National University of Singapore Institutional Review Board. Prior to randomization, all participants were required to complete a run-in period by wearing the activity tracker for ≥10 waking hours each day for ≥7 of the past 10 days.

### Randomization

Participants who completed the run-in period were randomized with equal probability into either of the two arms (Figure 1) using stratified randomization. Two stratification factors were used, (1) gender and (2) Fitbit®-logged activity levels during the 10-day run-in period with 2 levels: higher activity (at least 5 days with at least 10,000 steps) and lower activity (less than 5 days with at least 10,000 steps).

### Intervention

The incentive amount was set at SGD50 (≈USD37) per month, which was deemed large enough to induce a change in behavior but not so large as to be perceived as unaffordable by prospective payers, which could include employers or governments. Thus, the maximum payout possible was SGD200 (≈USD148) over 4 months. No penalty was incurred if a monthly step target was not met. Instead, participants were encouraged to try again in the subsequent month.

All participants were provided with the wrist-worn Fitbit® Inspire 3 activity tracker upon enrolment but prior to randomization. This tracker automatically links with the Fitbit® app and website. Participants were instructed to try to attain 10,000 steps daily. We selected 10,000 steps/day as a target for the intervention given that it is a simple and commonly supported step-based recommendation (22, 23) that is also displayed on the Fitbit® app and website. To obtain the incentives, we originally set the monthly target to meet this goal on at least 20 days out of the first 28 days of a month. We subsequently increased this target to 25 days a month for the final 190 participants as early data showed that the majority of participants (76.7%) in both arms were meeting the step target, thus reducing the ability to test our hypotheses (Supplementary Table S1).

Participants randomized into the cash arm were awarded SGD50 (≈USD37) each month they met the monthly step target. Those who met the step target were instructed to submit a claim for their incentive payment. This amount was transferred directly into their bank account at month’s end or early the next month. Participants randomized into the hedonic arm were reimbursed up to this level for expenses on hedonic activities if they met the same step target. Participants were provided with a list of hedonic incentive options to choose from before the start of each monthly cycle. These included (1) movie tickets and associated expenses (e.g., food and beverages purchased at cinemas), (2) karaoke, (3) manicure / pedicure, (4) massage, (5) dining / food delivery, (6) spa, (7) theme parks and other attractions, (8) video games and associated expenses (e.g., gaming devices and in-game currency), (9) vacation, (10) concerts / musical performances, and (11) other pleasurable experiences which were subject to the research team’s approval. Activities or substances that are known to put one’s health and wellbeing at risk (e.g., cigarettes, alcohol, gambling), and non-hedonic items (e.g., groceries, consumables) were not eligible. Those who met the step target in the hedonic arm were instructed to submit a claim for their chosen incentive along with a receipt showing proof of expenditure. The claimed amount, up to a maximum of SGD50 (≈USD37), was then reimbursed into their bank account. At any point during the trial, participants in both arms could check their monthly steps progress, claim status, and account balance on our study’s website.

### Outcomes and assessments

PA outcomes were tracked via the Fitbit®. The primary outcome was defined as the mean proportion of months that participants met the step target out of the 4 months. We chose 4 months as this was the longest duration possible that would allow for testing our hypotheses and being within the available budget. We also assessed (1) mean daily steps and (2) mean fairly and very active minutes as determined by Fitbit®‘s proprietary algorithms. We administered the Global Physical Activity Questionnaire (GPAQ) (24) developed by World Health Organization (WHO) to assess mean daily minutes spent in sedentary behavior and in three domains (1) activity at work, (2) travel to and from places, and (3) recreational activities. This was administered to identify which domain any identified step increase may be coming from. We additionally administered a modified version of the 8-item Physical Activity Enjoyment Scale (PACES) (25) to explore how incentives may impact the experience of engaging in PA. Self-determination theory posits that offering an incentive may undermine the enjoyment of PA (26, 27). This version of the PACES assesses enjoyment of PA on a 7-point scale (1 – *Strongly disagree*, 7 – *Strongly agree*). Scores on all 8 items are summed and averaged, resulting in a score range of 1 to 7 whereby higher scores indicate higher levels of enjoyment. Both the GPAQ and PACES have been validated in Asian populations (28, 29).

### Sample size calculation

Data on the variability in the proportion of months meeting the step target (primary outcome) in the presence of incentives is lacking. Therefore, this study was powered to detect a small-to-medium standardized effect size difference of 0.33 (mean difference divided by the pooled standard deviation) in the mean value of the primary outcome between the cash incentive and hedonic incentive arms, with a 80% statistical power and a 5% (two-sided) significance level (30), requiring 292 participants (146 in each arm). The study planned to recruit 310 participants to account for potential attrition.

### Statistical analyses

Analyses were conducted on an intention-to-treat basis. Firstly, differences in demographic outcomes between the cash and hedonic incentive arms were assessed using t-test and chi-square tests. We employed generalized linear regressions with identity link function and appropriate distribution function (normal distribution for continuous outcomes and binomial distribution for binary outcomes) to compare outcomes between the cash and hedonic incentive arms, while controlling for gender and baseline PA activity levels (greater or less than 5 days with at least 10,000 steps). Treatment effects, including the proportion of months step target was achieved, daily steps, and daily Fitbit® fairly and very active minutes, were calculated based on activity data pooled over 4 months. Additional sensitivity analyses were performed by imputing missing data for primary and secondary outcomes using the multiple imputation technique with 20 iterations to assess the impact of missing records on the comparison between two arms.

## Results

### Sample

Figure 1 presents the CONSORT diagram. Overall, 667 participants completed the screener; 310 were eligible and randomly assigned to the two arms. On average, participants were 43.2 years old (SD = 12.9) and 45.8% were male. Most participants (42.9%) reported a monthly household income of ≥ SGD7,000 (≈USD 5,135), were university graduates (76.8%), of Chinese ethnicity (93.9%), and married (52.3%) (Table 1). In comparison, the population in Singapore has a larger percentage of non-Chinese, a lower income, and is less educated (31, 32).

**Table 1:** Participant demographic measures by study arm.

Outcomes		Total	Cash arm	Hedonic arm	*p*
*N*		310	154	156	
Age, Mean (SD)		43.2 (12.9)	42.6 (13.3)	43.9 (12.5)	0.39^a^
Gender (%)					
	Male	45.8%	45.5%	46.2%	0.90^b^
Ethnicity (%)					
	Chinese	93.9%	94.2%	93.6%	0.83^b^
	Others	6.1%	5.8%	6.4%	
Marital status (%)					
	Married	52.3%	48.7%	55.8%	0.34^b^
	Widowed/ Separated from spouse/ Divorced/Prefer not to say	5.2%	6.5%	3.8%	
	Never married	42.6%	44.8%	40.4%	
Highest education level (%)					
	Up to secondary	6.1%	5.2%	7.1%	0.58^b^
	Post-secondary	17.1%	15.6%	18.6%	
	University bachelor’s degree/postgraduate diploma/degree	76.8%	79.2%	74.4%	
Household income (%)					
	Below SGD 4,000	19%	20.1%	17.9%	0.93^b^
	SGD 4,000–6,999	18.4%	16.9%	19.9%	
	SGD 7,000–9,999	15.8%	14.9%	16.7%	
	SGD 10,000 and over	27.1%	27.9%	26.3%	
	Prefer not to say/Do not know	19.7%	20.1%	19.2%	
Have you ever used a sport and fitness app to track your physical activities? (%)					
	Yes – Using the app now	78.1%	77.3%	78.8%	0.58^b^
	Yes – Used the app before but no longer using	16.5%	18.2%	14.7%	
	No	5.5%	4.5%	6.4%	

^a^*p*-value results based on a *t*-test.

^b^*p*-value results based on a chi-square test.

During the run-in period prior to randomization, PA levels were similar between arms. Participants had a mean of 11,900 (SD = 4,400) and 11,700 (SD = 4,050) steps per day in the cash and hedonic arms, respectively (Table 2). In total, 55.2 and 53.2% were in the higher activity group, exceeding 10,000 steps on at least 5 out of 7 days during the run-in period. Participants had a mean of 59.3 and 56.8 Fitbit® fairly and very active minutes a day in the cash and hedonic arms, respectively. These findings suggest that participants are far more active than the typical adult in Singapore (17). There were no significant differences between the arms for any of the demographic variables.

**Table 2:** Baseline physical activity levels and enjoyment by study arm.

Outcomes, Mean (SD)	Cash arm (*N* = 154)	Hedonic arm (*N* = 156)
High baseline physical activity level (at least 5 days with at least 10,000 steps), *N* (%)	85 (55.2%)	83 (53.2%)
Mean daily steps (in ‘000)	11.9 (4.4)	11.7 (4.05)
Mean daily Fitbit® fairly and very active minutes	59.3 (40.07)	56.8 (37.90)
^a^Total physical activity (minutes per day)	84.3 (94.17)	79.1 (94.39)
^a^Activity at work (minutes per day)	12.6 (37.25)	14.9 (41.51)
^a^Travel to and from places (minutes per day)	37.9 (54.76)	35.0 (51.96)
^a^Recreational activities (minutes per day)	33.8 (37.88)	29.2 (42.71)
^a^Sedentary activities (minutes per day)	442.6 (206.50)	441.4 (202.04)
^b^Physical activity enjoyment	4.9 (1.28)	4.7 (1.16)

^a^As measured by GPAQ = Global Physical Activity Questionnaire.

^b^As measured by PACES = 8-item Physical Activity Enjoyment Scale.

### Effect of the incentives on primary and secondary outcomes

As shown in Table 3, participants in both arms increased their mean daily steps during the intervention period compared to baseline. The mean increase was 870 steps (95% CI: 360, 1,370; *p* < 0.001) in the cash arm and 1,000 steps (95% CI: 520, 1,490; *p* < 0.001) in the hedonic arm. Similarly, mean daily Fitbit® fairly and very active minutes increased by 5.86 min (95% CI: 1.66, 10.06; *p* < 0.001) and 9 min (95% CI: 4.71, 13.29; *p* < 0.01) among participants in the cash and hedonic arms, respectively. For both arms, participant responses on the GPAQ suggest that physical activity minutes per day increased in all domains. Only for the travel domain in the cash arm was this increase not statistically significant. Furthermore, while both the cash (mean change from baseline: *β* = −24.84; 95% CI: −55.29, 5.61; *p* < 0.11) and hedonic arms (*β* = −20.32; 95% CI: −51.48, 10.84; *p* < 0.20) experienced a decrease in minutes spent in sedentary activities, this decrease did not reach statistical significance. Contrary to what self-determination theory would predict, both the cash (*β* = 0.34; 95% CI: 0.13, 0.55; *p* < 0.01) and hedonic arm participants (*β* = 0.40; 95% CI: 0.19, 0.62; *p* < 0.001) reported an increase in enjoyment of physical activity by the end of the intervention.

**Table 3:** Mean (95% CI) in physical activity levels and enjoyment over 4 months.

Outcomes	Cash arm (*N* = 154)	Hedonic arm (*N* = 156)	Difference^a^ (Hedonic Arm-Cash Arm)	SES^a^
Proportion of months step target was achieved	90.53 (86.87, 94.20)	88.34 (84.71, 91.98)	−2.19 (−7.33, 2.95)	−0.10
^b^Change in daily steps from baseline (in ‘000)	0.87*** (0.36, 1.37)	1.00*** (0.52, 1.49)	−0.02 (−0.49, 0.44)	−0.01
^b^Change in daily Fitbit® fairly and very active minutes from baseline	5.86** (1.66, 10.06)	9.00***(4.71, 13.29)	0.87 (−4.04, 5.78)	0.04
^b,c^Change in total physical activity from baseline (minutes per day)	31.04** (12.01, 50.08)	44.29*** (23.86, 64.73)	9.14 (−16.94, 35.21)	0.08
^b,c^Change in activity at work from baseline (minutes per day)	16.71** (5.23, 28.19)	10.19* (0.82, 19.56)	−3.81 (−18.71, 11.08)	−0.06
^b,c^Change in travel to and from places from baseline (minutes per day)	3.79 (−6.52, 14.11)	17.62*(3.02, 32.23)	11.40 (−4.27, 27.06)	0.16
^b,c^Change in recreational activities from baseline (minutes per day)	10.54** (2.78, 18.30)	16.48** (6.54, 26.42)	1.55 (−10.23, 13.33)	0.03
^b,c^Change in sedentary activities from baseline (minutes per day)	−24.84 (−55.29, 5.61)	−20.32 (−51.48, 10.84)	2.49 (−38.45, 43.43)	0.01
^b,d^Change in physical activity enjoyment from baseline	0.34** (0.13, 0.55)	0.40*** (0.19, 0.62)	−0.13 (−0.39, 0.13)	−0.11

CI, confidence interval; SES, Standardized effect size.

^a^Between arm comparison results based on a generalized linear model adjusted for baseline activity level and gender.

^b^Within arm comparison results are based on the paired t-test.

^c^As measured by GPAQ = Global Physical Activity Questionnaire.

^d^As measured by PACES = 8-item Physical Activity Enjoyment Scale.

*p < 0.05, **p < 0.01, ***p < 0.001.

However, between-arm comparisons reveal no significant difference for any of the outcomes assessed (Table 3). Sensitivity analysis revealed that the pattern of findings remained unchanged when the model used data with missing records imputed with the multiple imputation technique (Supplementary Table S2).

## Discussion

This study investigated the effectiveness of hedonic versus cash incentives in increasing PA levels using a randomized controlled trial design. We found that both strategies were very effective at increasing activity levels when compared to baseline. While these results substantiate the effectiveness of cash and hedonic incentives, between-arm comparisons reveal no meaningful difference for any of the outcomes assessed. Thus, we cannot claim one approach to be superior.

There are several reasons that could explain the lack of meaningful differences across arms. The most likely could be that individuals do not differentially value these incentive types despite the theory suggesting otherwise. At baseline, when participants were asked to rank their top three preferred incentive choices from a list of tangible and non-tangible incentives with utilitarian or hedonic qualities, the majority chose ‘cash payouts’, ‘vouchers for groceries’, and ‘vouchers for transportation expenses’ in that order (Supplementary Table S3). Other populations have shown a similar preference (32, 33). This suggests a general preference for incentives that have more utilitarian value. Although preferences do not necessarily predict outcomes, offering an incentive that is less preferred by participants could dampen its effectiveness (11). Feedback from participants in the hedonic arm revealed that many did not use the incentives for incremental activities but merely saw them as a way to receive funds for purchases they would have made regardless. This too diminishes the effective of the hedonic incentive strategy.

It is also possible that limitations with our design diluted the effectiveness of the hedonic arm. Unlike cash arm participants, those in the hedonic arm had to spend their own money before being reimbursed. This additional barrier could have created a disincentive but was required by our accounting office to ensure funds were not misallocated. Although possible, only a small number of participants (4.5%) who met the step goal did not submit a claim (Supplementary Table S4). The large increases from baseline coupled with the number of participants (86.5%) who met the step goal and submitted a claim suggests this is unlikely to be driving the results.

Furthermore, our study may have been underpowered to detect differences across arms. Given the high success rate observed early in the study, in efforts to generate greater variability in the primary outcome, we raised the incentive target from 20 to 25 days a month of 10,000 or greater steps. Although this change reduced the percentage of participants who met the monthly target, as expected, we again observe no meaningful difference between arms (Supplementary Table S1). Additionally, given that participants in this study were found to be more active than the typical adult in Singapore (18), our sample may exhibit selection bias as individuals who are more physically active may have been more likely to enroll into the study. Hence, caution should be exercised when generalizing our study’s results.

Given that individuals in both arms greatly increased their step activity during the intervention period, and differences between arms were small and with overlapping confidence intervals, the most likely conclusion is that both strategies are effective, and neither is clearly superior to the other. This finding is consistent with results from a literature review that compares several behavioral economic incentive strategies (e.g., lotteries, deposit contracts) versus cash incentives (13). Although that review did not include hedonic incentives and studies varied along multiple dimensions (e.g., duration, size of incentives, location), their general conclusion was these strategies are effective, at least in the short run, and, as in our case, no single strategy outperformed the others. Our results are also consistent with prior studies that have tested the effectiveness of cash vs. hedonic incentives in other domains, including employee performance and experimental tasks (14 (Tournament 1), 33, 35). These studies also found no differences in effectiveness.

It is worth noting that contrary to what self-determination theory would predict (26, 27), both the cash and hedonic arm participants reported an increase in enjoyment of physical activity at study conclusion based on the PACES scores. Thus, there appears to be no undermining effect of the incentives. This finding is important as increased enjoyment is predictive of habit formation and maintenance (25), thus it is possible that the behaviors could be sustained even after the incentives are removed. Past studies have also suggested that removing hedonic incentives could be less detrimental to performance (17, 35). Testing differential responses to incentive removal could be an area of future research.

Additionally, a limitation of our study is that participants were of higher income and more highly educated than the typical Singaporean. It is possible that cash and hedonic incentives could have differential effectiveness for lower income populations (12). These individuals have less disposable income such that the marginal value of cash may be greater for them. However, they may also experience fewer hedonic pleasures, such that the hedonic arm may be more effective for them. Testing the differential effectiveness of the two interventions in lower income populations could also be an area of future research.

Lastly, future studies may benefit from examining clinical health improvements associated with increases in physical activity. Although establishing clinical health improvements was not the focus of this study, the significant increases in daily steps of 870 steps and 1,000 steps within the cash and hedonic incentive arms, respectively, are comparable to clinically significant levels of step increments (36, 37). Indeed, increases in daily steps of up to 15,000 steps and 20,000 steps have been shown to exhibit an inverse relationship with all-cause and cardiovascular mortality (36). Specifically, every 500-step increment was associated with a 15% decreased risk of all-cause mortality, whereas a 1,000-step increment was associated with a 7% decreased risk of cardiovascular mortality (36). Therefore, examining clinical health improvements in future studies may lend more compelling evidence to justify investments toward incentivized physical activity programs.

Notwithstanding, the initial evidence drawn from the findings of this study suggest that hedonic incentives can be a viable and potentially more cost-effective alternative to cash incentives. Although we had set SGD50 (≈USD37) as the maximum hedonic incentive amount to allow participants greater flexibility and freedom to engage in a variety of hedonic activities, hedonic rewards can often be purchased at lower cost than their cash equivalent when purchased in bulk (e.g., spa packages). Therefore, funders of physical activity programs may be able to offer a SGD50-like experience without needing to spend the same amount, making hedonic incentives a more cost-effective and sustainable option than cash incentives. Future programs could leverage on hedonic incentives in designing physical activity programs that are more likely to enhance enjoyment and engagement over time by catering to a wide spectrum of preferences and motivations.

## Conclusion

This study demonstrated the effectiveness of cash and hedonic incentives to increase activity levels over a 4-month period. Individuals in both arms greatly increased their step activity during the intervention period relative to baseline. Differences between arms were small and not statistically significant, suggesting that either strategy represents a viable approach for increasing activity levels.

## Data availability statement

The raw data supporting the conclusions of this article will be made available by the authors, without undue reservation.

## Ethics statement

The studies involving humans were approved by National University of Singapore Institutional Review Board (Approval Number: NUS-IRB-2020-65). The studies were conducted in accordance with the local legislation and institutional requirements. The participants provided their written informed consent to participate in this study.

## Author contributions

EF: Investigation, Project administration, Supervision, Validation, Visualization, Writing – original draft, Writing – review & editing, Conceptualization, Funding acquisition, Methodology, Resources. MC: Data curation, Formal analysis, Investigation, Methodology, Project administration, Resources, Supervision, Validation, Visualization, Writing – original draft, Writing – review & editing. MG: Formal analysis, Resources, Visualization, Writing – review & editing.
