# Supplementary material for: Are cash incentives always king? A randomized controlled trial evaluating hedonic versus cash incentives (TEH-C)
Source: Front Public Health. 2024 Apr 30;12:1354814. doi: 10.3389/fpubh.2024.1354814 (PMC11091446; doi:10.3389/fpubh.2024.1354814)
Supplement: Supplementary file 1 [file Data_Sheet_1.docx]

Supplementary Material

**Table S1** Participants who met the 20-day and 25-day step target for 4 months by study arm.

|  | Cash Arm | | Hedonic Arm | |
| --- | --- | --- | --- | --- |
| Outcomes | Met Step Target | Total *N* | Met Step Target | Total *N* |
| 20-day step target | 46 (76.7%) | 60 | 46 (76.7%) | 60 |
| 25-day step target | 74 (78.7%) | 94 | 70 (72.9%) | 96 |

**Table S2** Mean (95% CI) in physical activity levels over 4 months^a^.

| Outcomes | Cash Arm  (*N* = 154) | Hedonic Arm  (*N* =156) | Difference^b^ (Hedonic Arm  *vs*  Cash Arm) | SES^b^ |
| --- | --- | --- | --- | --- |
| Proportion of months step target was achieved | 95.23  (92.73, 97.73) | 94.40  (91.97, 96.83) | -0.83  (-4.29, 2.62) | -0.06 |
| Daily steps (in ‘000) | 12.75  (12,45, 13.05) | 12.78  (12.48, 13.08) | 0.02  (-0.40, 0.45) | 0.01 |
| Daily Fitbit^®^ fairly and very active minutes | 65.73  (62.40, 69.06) | 66.98  (63.67, 70.28) | 1.24  (-3.43, 5.92) | 0.06 |

CI = confidence interval; SES = Standardised effect size

^a^Sensitivity analyses were performed by imputing missing data using the multiple imputation technique with 20 iterations.

^b^Between arm comparison results based on a generalised linear model adjusted for baseline activity level and gender.
*Note:* There were no missing records for both the Global Physical Activity Questionnaire (GPAQ) and 8-item Physical Activity Enjoyment Scale (PACES) measured outcomes, so sensitivity analyses were not conducted for these outcomes.

* *p* < 0.05, ** *p* < 0.01, *** *p* < 0.00

**Table S3** Top 3 preferred reward choices at baseline.

| Outcomes | | Cash Arm  (*N* = 154) | Hedonic Arm  (*N* =156) |
| --- | --- | --- | --- |
| Baseline Type of Reward Choice (%) | |  |  |
|  | Cash payouts | 148 (96.1%) | 148 (94.9%) |
|  | Vouchers for groceries | 135 (87.7%) | 134 (85.9%) |
|  | Vouchers for transportation expenses | 72 (46.8%) | 69 (44.2%) |
|  | Vouchers for utility bills | 41 (26.6%) | 51 (32.7%) |
|  | Vouchers for movie tickets | 13 (8.4%) | 15 (9.6%) |
|  | Vouchers for overseas holiday expenses | 13 (8.4%) | 12 (7.7%) |
|  | Donations to a charity of your choice | 12 (7.8%) | 9 (5.8%) |
|  | Vouchers for spa or massages | 9 (5.8%) | 10 (6.4%) |
|  | Vouchers for healthcare or medical expenses | 7 (4.5%) | 8 (5.1%) |
|  | Other | 12 (7.8%) | 12 (7.7%) |

**Table S4** Type of hedonic incentive claimed by month.

| Outcomes | | Month 1 | Month 2 | Month 3 | Month 4 |
| --- | --- | --- | --- | --- | --- |
| Hedonic Reward Type (%) | |  |  |  |  |
|  | Dining / Food delivery | 106 (67.9%) | 117 (75.0%) | 118 (75.6%) | 116 (74.4%) |
|  | Movie tickets and associated expenses | 12 (7.7%) | 2 (1.3%) | 1 (0.6%) | 1 (0.6%) |
|  | Massage | 8 (5.1%) | 5 (3.2%) | 4 (2.6%) | 5 (3.2%) |
|  | Spa (body / facial) | 3 (1.9%) | 3 (1.9%) | 2 (1.3%) | 1 (0.6%) |
|  | Theme parks and other attractions | 3 (1.9%) | 0 | 1 (0.6%) | 0 |
|  | Vacation | 3 (1.9%) | 6 (3.8%) | 3 (1.9%) | 1 (0.6%) |
|  | Video games and associated expenses | 2 (1.3%) | 3 (1.9%) | 2 (1.3%) | 1 (0.6%) |
|  | Concerts / Musical Performances | 1 (0.6%) | 0 | 0 | 0 |
|  | Karaoke | 0 | 1 (0.6%) | 0 | 0 |
|  | Manicure / pedicure | 0 | 0 | 1 (0.6%) | 0 |
|  | Other pleasurable experiences (subject to approval) | 4 (2.6%) | 3 (1.9%) | 0 | 0 |
| Missing approved claims | |  |  |  |  |
|  | Not eligible for the incentive | 11 (7.1%) | 9 (5.8%) | 19 (12.2%) | 17 (10.9%) |
|  | Eligible for the incentive^a^ but did not submit any claims | 3 (2.0%) | 7 (4.5%) | 5 (3.2%) | 13 (8.3%) |
|  | Eligible for the incentive^a^ but there are only rejected claims | 0 | 0 | 0 | 1 (0.6%) |

^a^Eligible for the incentive indicates that the participant has met the step target for the month.

**Screener Questionnaire**

Thank you for your interest in the TEH-C research study. We would like you to complete a short survey so that we can determine whether you are eligible to participate in this study. Please answer the following questions carefully and to the best of your knowledge. Your answers will be kept strictly confidential.

This screener survey will take approximately 3 minutes or less to complete.

1. How old were you at your last birthday? (Numerical)
2. Are you currently residing in Singapore with no plans to relocate during your participation in this 4-month study? (Yes/No)
3. Do you use your phone to perform the following on a regular basis? (Yes/No)
   1. Send and receive emails
   2. Take photos
   3. Browse the Internet
   4. Install apps on your phone
4. This study consists of various reward interventions and you will be randomly allocated into one of the intervention groups, also known as study arms. This means that you cannot choose which study arm you will be assigned to and cannot change study arms at any point in the study. All participants, regardless of which study arm they are allocated to, will be given the same physical activity goals and will have equal opportunity to earn up to S$200 worth of rewards in total over 4 calendar months. Are you willing to be randomly allocated to an intervention group (study arm)? (Yes/No)
5. A pedometer (e.g., Fitbit^®^ physical activity tracker) is a small step counter worn on the wrist that counts that number of steps you walk/run. The research team will use data collected from your pedometer to analyze your activity levels. Are you willing to wear a pedometer every day for four calendar months during waking hours? (Yes/No)
6. Are you a woman who is currently pregnant or lactating? (Yes/No)
7. Are you able to walk up 10 steps (individual steps, not floors) without stopping? (Yes/No)
8. Are you currently advised by your doctor that you should not engage in moderate-to-vigorous physical activity (i.e., brisk walking or more intense)? (Yes/No)
9. Do you have any condition that restricts you from engaging in moderate-to-vigorous physical activity (i.e., brisk walking or more intense)? (Yes/No)

**
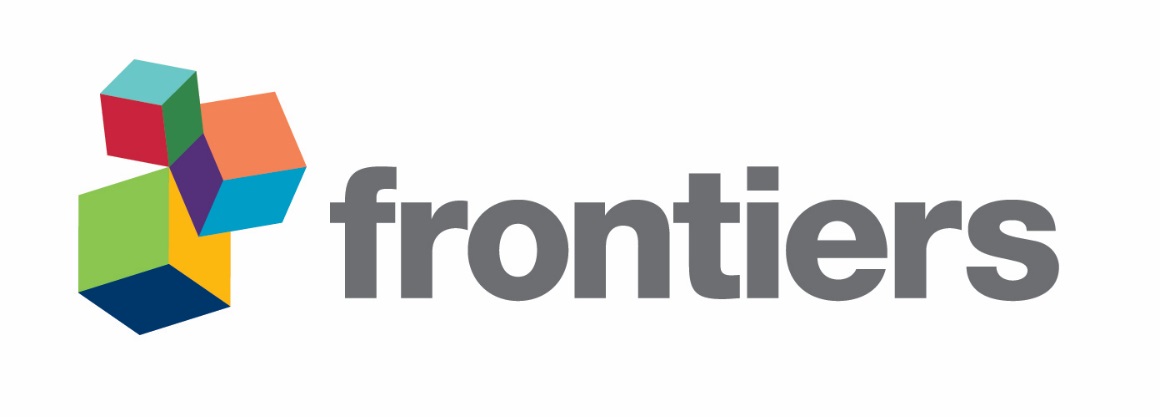
**
